# Study of The Molecular Nature of Congenital Cataracts in Patients from The Volga–Ural Region

**DOI:** 10.3390/cimb45060327

**Published:** 2023-06-15

**Authors:** Irina Khidiyatova, Indira Khidiyatova, Rena Zinchenko, Andrey Marakhonov, Alexandra Karunas, Svetlana Avkhadeeva, Marat Aznzbaev, Elza Khusnutdinova

**Affiliations:** 1 Institute of Biochemistry and Genetics—Subdivision of the Ufa Federal Research Centre of the Russian Academy of Sciences, 450054 Ufa, Russia; indi-86@mail.ru (I.K.); carunas@list.ru (A.K.); elzakh@mail.ru (E.K.); 2 Department of Biology, Bashkir State University, 450076 Ufa, Russia; 3 Research Centre for Medical Genetics, 115522 Moscow, Russia; renazinchenko@mail.ru (R.Z.); marakhonov@generesearch.ru (A.M.); 4 Medical Faculty, Bashkir State Medical University, 450000 Ufa, Russia; avhadeeva-s@mail.ru (S.A.);

**Keywords:** hereditary congenital cataract, crystallin genes *CRYAA*, *CRYAB*, *CRYGC*, *CRYGD*, *CRYBA1*, connexin genes *GJA8*, *GJA3*, Volga–Ural Region

## Abstract

Hereditary cataracts are characterized by significant clinical and genetic heterogeneity, which can pose challenges for early DNA diagnosis. To comprehensively address this problem, it is essential to investigate the epidemiology of the disease, perform population studies to determine the spectrum and frequencies of mutations in the responsible genes, and examine clinical and genetic correlations. Based on modern concepts, non-syndromic hereditary cataracts are predominantly caused by genetic disease forms associated with mutations in crystallin and connexin genes. Therefore, a comprehensive approach to studying hereditary cataracts is necessary for early diagnosis and improved treatment outcomes. The crystallin (*CRYAA*, *CRYAB*, *CRYGC*, *CRYGD,* and *CRYBA1*) and connexin (*GJA8*, *GJA3*) genes were analyzed in 45 unrelated families from the Volga–Ural Region (VUR) with hereditary congenital cataracts. Pathogenic and probably pathogenic nucleotide variants were identified in ten unrelated families, nine of which had cataracts in an autosomal dominant pattern of inheritance. Two previously undescribed likely pathogenic missense variants were identified in the *CRYAA* gene: c.253C > T (p.L85F) in one family and c.291C > G (p.H97Q) in two families. The known mutation c.272_274delGAG (p.G91del) was found in the *CRYBA1* gene in one family, while no pathogenic variants were found in the *CRYAB*, *CRYGC*, or *CRYGD* genes in the examined patients. In the *GJA8* gene, the known mutation c.68G > C (p.R23T) was found in two families, and previously undescribed variants were identified in two other families: a c.133_142del deletion (p.W45Sfs*72) and a missense variant, c.179G > A (p.G60D). In one patient with a recessive form of cataract, two compound-heterozygous variants were identified—a previously undescribed likely pathogenic missense variant, c.143A > G (p.E48G), and a known variant with uncertain pathogenetic significance, c.741T > G (p.I24M). Additionally, a previously undescribed deletion, c.del1126_1139 (p.D376Qfs*69), was identified in the *GJA3* gene in one family. In all families where mutations were identified, cataracts were diagnosed either immediately after birth or during the first year of life. The clinical presentation of the cataracts varied depending on the type of lens opacity, resulting in various clinical forms. This information emphasizes the importance of early diagnosis and genetic testing for hereditary congenital cataracts to guide appropriate management and improve outcomes.

## 1. Introduction

Cataracts are a leading cause of blindness and are characterized by the presence of light-scattering opacities in the lens of the eye. Congenital or infantile cataracts, which present within the first year of life, account for up to 10% of childhood blindness cases worldwide. The prevalence of hereditary congenital isolated (non-syndromic) cataracts is estimated to be between 1–6 per 10,000 children [1]. Early diagnosis and prompt surgical intervention are essential to prevent irreversible amblyopia and ensure normal visual development in children with congenital cataracts.

Hereditary congenital cataracts (HCCs) are phenotypically and genetically heterogeneous and are typically inherited in an autosomal dominant manner, but can also be inherited in an autosomal recessive or X-linked manner. To date, over 50 genes have been identified [2] that are responsible for the development of hereditary congenital cataracts. Mutations in genes encoding crystallins, which constitute up to 90% of all lens proteins and play a vital role in maintaining lens transparency and refractive index, are the most common cause of HCCs. Mutations in genes encoding connexins 50 and 46, which are structural proteins of intercellular gap junctions, are also a frequent cause of hereditary cataracts. These connexins facilitate the movement of small electrolytes and metabolites between lens cells [3].

The mutations detected in known genes among patients with hereditary cataracts living in different regions of the world are diverse. The accumulation of data on the population diversity of the spectrum of mutations and the corresponding phenotypic signs of cataracts is of considerable interest and contributes both to the improvement of methods of medical genetic counseling for families with this pathology and to the knowledge of the pathogenesis of the disease.

The main objective of our investigation was to comprehensively examine the epidemiological, clinical, genealogical, and molecular genetic characteristics of hereditary isolated congenital cataracts in the Bashkortostan Republic (BR). Our study aimed to enhance early diagnosis, prevention, and treatment of patients with hereditary cataracts. Bashkortostan is one of the multinational republics of the Volga–Ural region (VUR) of Russia, located in the foothills of the Southern Urals. The BR is a region of significant interest for investigating the epidemiology and molecular genetic basis of monogenic pathology given the unique genetic and demographic factors in this region, such as monoethnic marriages and high levels of endogamy that contribute to uneven distribution of hereditary diseases, including ophthalmological ones [4]. According to recent statistics, the prevalence of hereditary congenital cataracts in the BR is 0.84 per 10,000 people [5]. In this paper, we present the outcomes of our analysis of the crystallin genes αA (*CRYAA*), αB (*CRYAB*), γC (*CRYGC*), γD (*CRYGD*), βA1 (*CRYBA1*), and connexins 50 (*GJA8*) and 43 (*GJA3*) in patients with congenital isolated cataracts (CICs) from the Bashkortostan Republic of the VUR.

## 2. Materials and Methods

The present study involved the analysis of 94 patients with congenital isolated cataracts from 45 unrelated families, consisting of 16 Tatar, 14 Russian, 13 Bashkir, and 2 mestizo individuals residing in the Bashkortostan Republic. Clinical examination, including cataract extraction in most cases, was conducted at the Ufa Eye Disease Research Institute of Bashkir State Medical University (Russian Federation). The cataract diagnosis was established through conventional ophthalmological examination techniques, including visual acuity determination, biomicroscopy, refractometry, ophthalmoscopy, keratometry, echobiometry, and tonometry. All patients were confirmed to have an isolated form of cataract without any clinical manifestations of other conditions, such as neurological, cardiovascular, or musculoskeletal disorders. The type of cataract was determined through genealogical analysis within the patients’ families. Autosomal dominant inheritance was observed in 36 unrelated patients (80%), while autosomal recessive inheritance was seen in 3 patients (6.7%); the type of inheritance could not be precisely determined in 6 patients (13.3%).

To determine the clinical significance of the single nucleotide variants identified in the studied genes, we performed a screening analysis in healthy members of the probands’ families as well as in control individuals (185 people) from the Bashkortostan Republic of different ethnicities, including Russian (60 individuals), Tatar (65 individuals), and Bashkir (60 individuals).

The molecular genetic study was performed according to standard protocols. DNA was extracted from whole blood using the phenol–chloroform extraction method. The coding sequences of the *CRYAA* (Gene ID 1409; MIM123580, NG_009823.1), *CRYAB* (Gene ID 1410; MIM123590, NG_009824.2), *CRYGC* (Gene ID 1420; MIM604307, NG_008038.1), *CRYGD* (Gene ID 1421; MIM123690, NG_008039), *CRYBA1* (Gene ID 1411; MIM600881, NG_008037.1), *GJA8* (Gene ID 2703; MIM116200, NG_016242.1), and *GJA3*(Gene ID 2700; MIM601885, NG_ 016399.1) genes were amplified using specific primers that were either previously reported [6,7,8,9] or designed using Primer3 (v. 0.4.0) software (http://fokker.wi.mit.edu/primer3/input.htm (accessed on 10 January 2011). The sequences of the amplified coding regions were analyzed using an ABI PRISM 3500 XL Applied Biosystems automatic sequencer (Thermo Fisher Scientific Inc., Waltham, Massachusetts, USA). RFLP analysis was used to screen for the presence or absence of newly identified nucleotide variants in control samples.

The functional significance of the identified single nucleotide substitutions was assessed in silico using ten computer programs: PoliPhen2, v.2 (http://genetics.bwh.harvard.edu/pph2/), SIFT(http://sift.bii.a-star.edu.sg/), LRT(http://annovar.openbioinformatics.org), MutationTaster (http://www.mutationtaster.org), Mutation Assessor (http://mutationassesor.org), FATHMM (http://fathmm.biocompute.org.uk), PROVEAN (http://provean.jcvi.org), MetaLR (http://annovar.openbioinformatics.org), M-CAP (http://bejerano.stanford.edu/mcap), and CADD (http://cadd.gs.washington.edu) (Table 1). Pathogenicity of the nucleotide variants was interpreted following the recommendations of the American College of Medical Genetics (ACMG) [10].

Within a single family, next-generation sequencing (NGS) technology was utilized for the study. Sample preparation involved the selective capture of DNA regions that belonged to the coding regions of 4811 genes with known clinical significance, followed by sequencing using Illumina NextSeq 500, (Illumina, Inc., San Diego, CA, USA) via pair-ended reading (2 × 151 bp) with an average coverage of at least 70–100×. Automated algorithms were used for processing the sequencing data, including sequencing quality assessment, read alignment to the reference sequence of the human genome (GRCh37/hg19), post-processing, variant identification, and filtering by quality. The identified variants were annotated for all known transcripts of each gene from the RefSeq database using various methods for predicting the pathogenicity of substitutions, such as SIFT, PolyPhen2-HDIV, PolyPhen2-HVAR, MutationTaster, LRT, and BMut. Additionally, methods such as PhyloP and PhastCons were used for calculating the evolutionary conservation of positions. To estimate the population frequencies of the identified variants, databases such as the 1000 Genomes Project, ESP6500, ExAC, and gnomAD were used. The OMIM database and literature data were referred to for assessing the clinical relevance of the identified variants.

## 3. Results 

In this study, we investigated genetic variants in *CRYAA*, *CRYAB*, *CRYGC*, *CRYGD*, *CRYBA1*, *GJA8*, and *GJA3* genes in 94 patients with congenital isolated cataracts from 45 unrelated families. 

**Figure 1:**
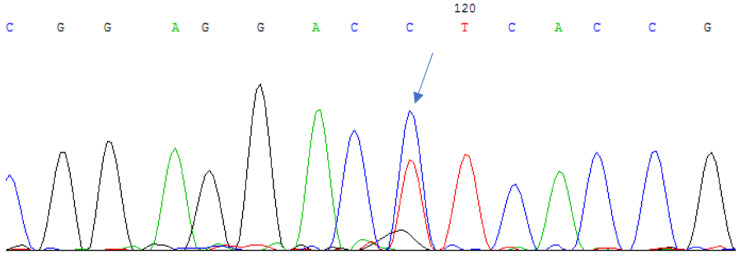
Sequencing of the second exon of the *CRYAA* gene in a patient with hereditary congenital cataract revealed a nucleotide substitution of c.253C > T (p.L85F) in the heterozygous state.

We identified a previously undescribed substitution of cytosine for thymine in the 253rd position of the nucleotide sequence in the second exon of the *CRYAA* gene in a family of Tatar ethnicity. This substitution was found in two siblings with autosomal dominant hereditary congenital cataracts in a heterozygous state, resulting in the substitution of leucine to phenylalanine at the 85th position of the amino acid sequence of the protein (c.253C > T (p.L85F)) (Figure 1).

At the time of our clinical genetic study, ophthalmological examination was available only for the two affected siblings, aged 6 and 8, who had undergone cataract surgery during early childhood (before 2 years of age). Initial examination revealed that both brothers had congenital bilateral nuclear cataracts, microphthalmos, and microcornea. During our study, they were also found to have nystagmus and convergent strabismus with a catalytic component in addition to these pathologies. The nucleotide substitution c.253C > T was confirmed by restriction analysis using AvaII endonuclease (New England Biolabs), which demonstrated the disappearance of the normal restriction site. This nucleotide substitution was not found in healthy members of the proband family or in the control sample of healthy individuals. The p.L85F variant was determined to have a high level of pathogenicity and a damaging effect on the structure and function of the protein using several predictive programs (see Table 1).

**Figure 2:**
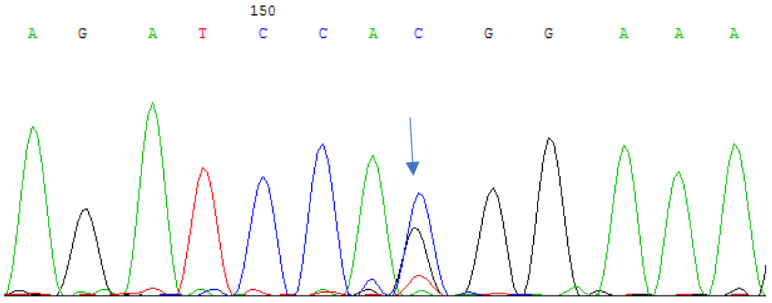
Sequencing of the second exon of the *CRYAA* gene in a patient with HCC: nucleotide substitution c.291C > G (p.H97Q) in the heterozygous state.

Two additional unrelated patients of Bashkir ethnicity were identified with a heterozygous substitution of cytosine for guanine at the 291st position of the nucleotide sequence in the second exon of the gene. This nucleotide substitution leads to the substitution of histidine for glutamine at the 97th position of the amino acid sequence of the protein (c.291C > G (p.H97Q), see Figure 2).

Both patients underwent cataract surgery in early childhood, with one being classified as “posterior polar cataract” while the inheritance type for the other could not be established. The latter patient had an autosomal dominant pattern of cataract inheritance and also suffered from lens subluxation. The c.291C > G nucleotide substitution, which leads to the substitution of histidine for glutamine at the 97th position of the amino acid sequence of the protein (p.H97Q), is absent from the databases used for analysis, but other extremely rare polymorphic variants have been recorded at this position (c.291C > T/A (p.H97Q) (rs144722442)), with a frequency of 0.00004 and 0.000004 in the total world sample, respectively, according to gnomAD. The results of functional significance analysis using predictive programs indicated a high probability of pathogenicity of the c.291C > G substitution (Table 1). The BstNI restriction enzyme can be used to detect the nucleotide substitution, which creates a restriction site that is absent in the normal sequence. This nucleotide substitution was not found in control samples of healthy individuals.

The *CRYAB* gene is associated with the development of cataracts, with 17 mutations identified as causative factors according to the CAT-MAP variant database (https://cat-map.wustl.edu/home/cat-map-variant-file/ (accessed on 20 February 2023)). In our study, a synonymous nucleotide change, c.165G > A (p.L55=), was identified in the *CRYAB* gene in the patients examined, which was a known neutral polymorphic variant (rs2228387) occurring in Eurasian populations at a frequency of 1–3%. This polymorphic variant was detected in five unrelated patients, four of whom were Bashkirs, and one a Ukrainian/Bashkir mestizo. Control samples of healthy individuals were not screened for the presence of this nucleotide substitution. No pathogenic variants in the *CRYAB* gene were found in the patients examined in our study.

**Figure 3:**
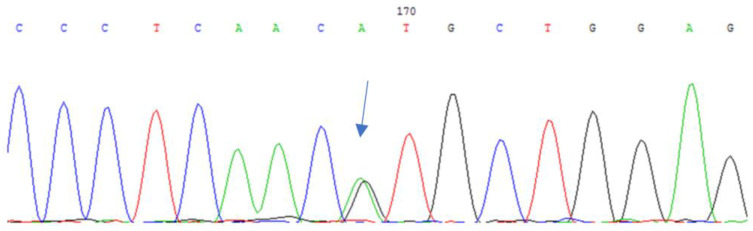
Sequencing of the third exon of the *CRYGD* gene in a patient with HCC: nucleotide substitution c.376G > A (p.V126M) in the heterozygous state.

In a patient of Russian ethnicity with congenital autosomal dominant nuclear cataract, a missense variant, c.376G > A (p.V126M) (rs150318966), was detected in the heterozygous state in the third exon of the *CRYGD* gene (Figure 3). Although most predictive programs suggest a high probability of pathogenicity for this variant (Table 1), it has been registered as a polymorphic variant in the gnomAD database with an allelic frequency of 0.004 in European populations. Additionally, homozygous carriers were also identified in the database, which makes it unlikely that this nucleotide substitution alone causes the development of a dominant form of the disease. The ClinVar database lists the rs150318966 variant as benign. We did not find the c.376G > A nucleotide substitution in our control samples of healthy individuals.

In a patient of Russian ethnicity, the missense variant c.130A > G (p.M44V) (rs140206746) was identified in the heterozygous state in the second exon of the *CRYGD* gene. According to predictive programs, this nucleotide substitution is a known benign polymorphic variant. In addition, in a patient of Tatar ethnicity, a rare neutral polymorphic variant in the 3’-noncoding sequence of the gene—c.*12T > C (rs2305429)—was found in the heterozygous state after studying the third exon of the gene. None of these nucleotide variants were found in our control samples of healthy individuals.

The *CRYGC* gene is associated with the development of cataracts, with 32 mutations identified as causative factors (https://cat-map.wustl.edu/home/cat-map-variant-file/ (accessed on 20 February 2023). In our sample of patients with cataracts, we did not observe any changes in the nucleotide sequence of the *CRYGC* gene.

**Figure 4:**
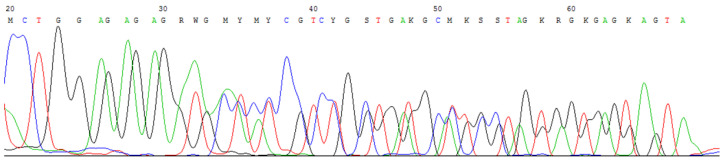
*CRYBA1* gene sequencing: heterozygous c.272_274delGAG (p.Gy91del) deletion in a patient with HCC.

Whole exome sequencing of one patient identified a heterozygous c.272_274delGAG (p.G91del) mutation in the *CRYBA1* gene, which was subsequently confirmed by Sanger sequencing (Figure 4). This mutation was also detected in two children of the proband, both of whom had cataracts in early childhood, and was absent in healthy family members, including one daughter and a sister.

In this Russian family, cataracts were observed in an autosomal dominant manner, affecting the proband (mother) and her two children, who all underwent cataract surgery in early childhood. Zonular cataracts were reported in their medical records. To assess the prevalence of the p.G91del mutation in other patients with hereditary cataracts from Brazil, 7% polyacrylamide gel electrophoresis was used, but no such mutation was detected in the 44 unrelated patients examined. No other pathogenic variants in the CRYBA1 gene were detected in this sample. However, two known variants in the gene were identified in the coding regions, which were assessed as benign polymorphic variants. Specifically, a missense variant—c.74C > T (p.P25L) (rs142631461)—was found in the second exon of the gene in a Bashkir patient and presented in the gnomAD database with an average allele frequency of 1.7%, 2.7% in the European population. In the fifth exon of 14 patients with different ethnicities, a synonymous substitution c.456C > T (rs1047790) was observed, with an allele frequency of 18.2% in the examined sample. The frequency of the rs1047790*T allele in the world averages 29%, varying from 17% in European populations to 36% in African and South Asian populations (1000 Genomes Project). Two neutral polymorphic variants were identified in the intron regions flanking the exons of the gene: rs2286407 (NC_000017.11: g.29249281t > g), with an allele frequency of 8.6%, and rs72819448 (NC_000017.11: g.292250316c > t), with an allele frequency of 1%. Overall, our examination of patients with hereditary cataracts detected a single pathogenic variant, c.272_274delGAG (p.G91del), in the *CRYBA1* gene, with a frequency of 2.2% in the total sample of patients and 3% among patients with autosomal dominant cataract.

**Figure 5:**
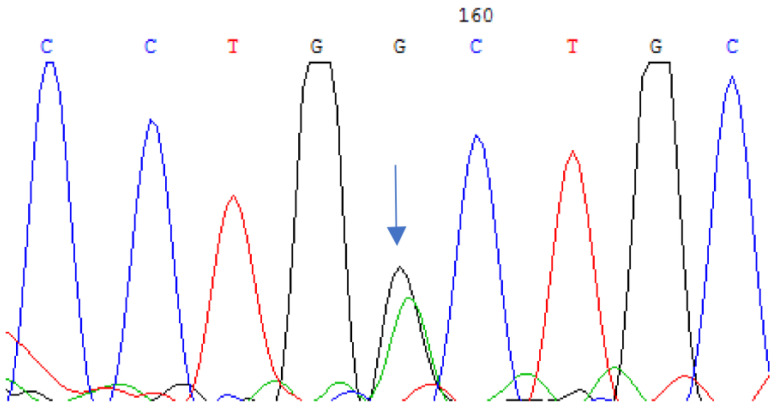
Sequencing of the *GJA8* gene: c.179G > A nucleotide substitution in the heterozygous state.

In this study, we identified pathogenic and probably pathogenic variants in the GJA8 gene in five unrelated families of patients with congenital isolated cataracts. Among patients of Russian ethnicity, we detected two unrelated individuals in the heterozygous state with the missense mutation c.68G > T(p.R23T) (rs80358203), previously reported in an Iranian family with hereditary autosomal dominant cataracts [11]. One patient presented with a punctate cataract, while the other had a nuclear cataract. The mother of one of these patients, who also had a history of congenital cataracts, was also found to carry this mutation. Unfortunately, we could not analyze the DNA of other family members. In three other unrelated patients, we identified previously undescribed nucleotide changes. In a patient of Russian ethnicity with congenital autosomal dominant zonular cataracts combined with microphthalmia and microcornea, we detected the heterozygous nucleotide substitution c.179G > A (p.G60D) (Figure 5). It is known that the patient’s father had a history of cataracts, but he was not available for clinical evaluation.

**Figure 6:**
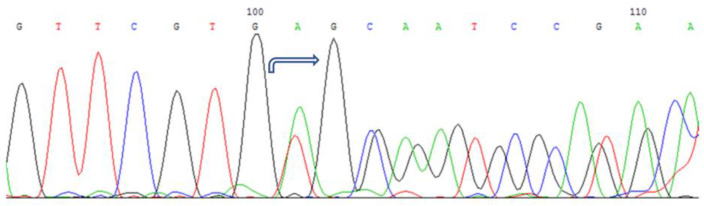
*GJA8* gene sequencing: c.133_142del deletion in the heterozygous state.

One patient of Bashkir ethnicity with anterior polar cataracts was found to have a deletion of ten nucleotides in the coding region of the *GJA8* gene in the heterozygous state, resulting in a frameshift and the creation of a premature stop codon (c.del133_142 (p.W45Sfs*72)) (Figure 6). However, the patient’s father, who also had a history of cataracts, was not available for further analysis of this family.

**Figure 7:**
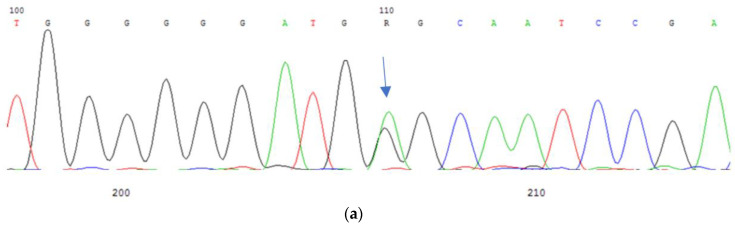

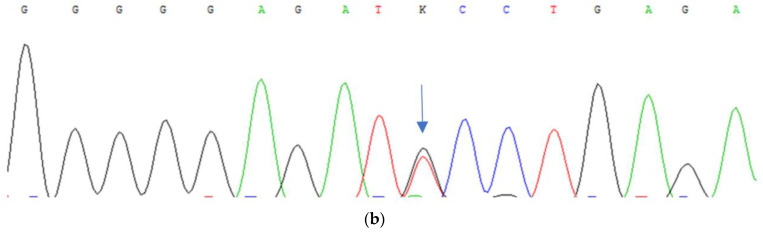
Sequencing of the *GJA8* gene: (**a**) nucleotide substitution c.143A > G (p.E48G) in the heterozygous state; (**b**) nucleotide substitution c.741T > G (p.I247M) in the heterozygous state.

In a Russian family, a patient with congenital bilateral zonular cataracts was found to have two nucleotide substitutions in the compound heterozygous state—a previously undescribed missense variant c.143A > G (p.E48G) and a well-known variant c.741T > G (p.I24M) (rs80358202) (Figure 7a,b). A clinical and molecular genetic study was conducted on three generations on the maternal and paternal lines. The nucleotide substitution c.143A > G was found to be maternally inherited (detected in the mother and grandmother), while the nucleotide substitution c.741T > G was paternally inherited (detected in the father and grandfather). Interestingly, the proband is the only affected member in the family, as carriers of the identified nucleotide substitutions, including both parents, maternal grandmother, and paternal grandfather, do not suffer from cataracts.

The newly identified nucleotide changes in the *GJA8* gene, namely c.179G > A, c.del133_142 and c.143A > G, were predicted to be pathogenic based on various predictive programs (see Table 1). None of these variants were detected in our control group of healthy individuals.

**Figure 8:**
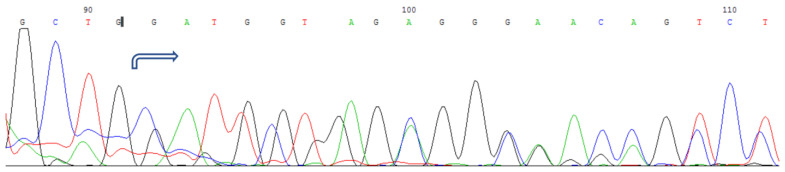
*GJA3* gene sequencing: c.del1126_1139 deletion in heterozygous state.

In a family of Tatar ethnicity with congenital autosomal dominant zonular cataracts, a deletion of 14 nucleotides was discovered in the *GJA3* gene c.del1126_1139 (p.D376Qfs*69) in the heterozygous state in three cataract patients of two generations (Figure 8). The deletion was absent in healthy family members as well as in the control group comprising healthy individuals, indicating its possible role as a pathogenic variant.

In addition to the pathogenic variants identified in the *GJA8* and *GJA3* genes, two rare polymorphic variants were found in the *GJA3* gene in two Bashkir families—c.231C > T (p.F77=) (rs143508620) and c.398G > A (p.R133Q) (rs149933083). These variants were predicted to be benign allelic variants by predictive programs and were not detected in our control groups. The minor allele frequency of c.231C > T and c.398G > A in the databases was 0.002 and 0.003, respectively, and they were also found in homozygous states, confirming their neutral character.

Thus, as a result of the study of seven genes in patients with congenital isolated cataracts from BR, 20 different changes in nucleotide sequences were identified, 6 of which were identified for the first time. Six nucleotide changes, according to predictive computer programs and based on their absence in healthy individuals, are probably pathogenic variants. Pathogenic and probably pathogenic variants were identified in ten unrelated families (22.2%); information about them is summarized in Table 2. In all families with identified mutations, cataracts were diagnosed immediately after birth or during the first year of life. In most cases, this was inherited by autosomal dominant type; the type of clouding of the lens had different clinical forms (Table 2).

## 4. Discussion

According to current understanding, non-syndromic hereditary cataracts are primarily caused by genetic mutations in the genes encoding crystallins and connexins. Crystallins are the largest contributors to lens proteins, comprising up to 90% of the total. They play a critical role in maintaining the lens’s refractive index [12]. In humans, three major classes of crystallin proteins have been identified: α-crystallins, which account for approximately 40%, β-crystallins, which comprise about 35%, and γ-crystallins, which make up 25% of the total crystallin content [13,14,15].

Alpha crystallins are large multimeric proteins, composed of αA and αB subunits that are encoded by *CRYAA* and *CRYAB* genes, respectively. Alpha-crystallins belong to the family of small heat shock proteins (sHSPs), which act as chaperones and mainly perform protective functions in the lens. They participate in the formation and dissociation of protein complexes, prevent incorrect binding of partially denatured proteins that accumulate in cells under the influence of harmful factors, and restore the correct tertiary or quaternary structure of proteins, including beta- and gamma-crystallins. This ensures the maintenance of lens transparency [16,17]. It is also known that alpha-crystallins protect cells from stress-induced apoptosis, regulate cell growth, and increase genome stability [18]. Gamma crystallins, encoded by the *CRYGD* and *CRYGC* genes, are the structural proteins of the lens, and play a crucial role in the development and maintenance of lens transparency [19]. Beta crystallins, on the other hand, remain the most poorly understood in their structural significance due to their greater number of subunits and possible oligomeric formations. The *CRYBA1* gene encodes two proteins, βA1- and βA3-crystallins, which are the most common water-soluble crystallins in the lens [20]. Various beta-crystallin proteins can interact with each other to form oligomers of different sizes, from dimers to octamers, and with other lens proteins. Protein–protein interactions play a key role in maintaining lens transparency, and mutations in crystallin genes that disrupt the structure and functions of the corresponding proteins can lead to the loss of lens transparency and the development of cataracts [21].

In our study of patients with congenital hereditary cataracts in the Bashkortostan Republic, we examined five crystallin genes, *CRYAA*, *CRYAB*, *CRYGC*, *CRYGD*, and *CRYBA1,* and two connexin genes, *GJA8* and *GJA3*. In the crystallin genes studied, pathogenic and potentially pathogenic nucleotide variants were discovered only in the *CRYAA* and *CRYBA1* genes. In the *CRYAA* gene, we found two previously undescribed missense variants, c.253C > T(p.L85F) and c.291C > G (p.H97Q), located in the second exon of the gene. The second and third exons of the *CRYAA* gene encode domains of the protein molecule homologous to small heat shock proteins, which play a crucial chaperone function in alpha-crystallin [22,23,24]. With the c.253C > T substitution, an uncharged leucine is replaced with a phenylalanine, which is a heterocyclic amino acid. With the c.291C > G substitution, the positively charged histidine, which has a heterocyclic side radical in physiological solutions, is replaced with an uncharged glutamine, an aliphatic amino acid with a hydrophilic protein radical. These amino acid substitutions can potentially change the conformational structure of the protein and disrupt its chaperone function, leading to cataract development. Our in silico analysis using predictive programs supports the likelihood that these nucleotide substitutions are pathogenic variants leading to congenital cataracts.

Previous studies have investigated the *CRYAA* gene in cataract patients from various countries worldwide. Currently, there are 26 different pathogenic variants identified in this gene (https://cat-map.wustl.edu/home/cat-map-variant-file/ (accessed on 20 February 2023)). These mutations are predominantly located in the N-terminal and α-crystallin domains of the protein; several pathogenic variants have been identified in the C-terminal domain and most of them are missense mutations, although cases with nonsense, microdeletion, and splice-site mutations have also been reported. The mutations in the amino acid sequence of the CRYAA protein often affect arginine residues and cause a dominant form of cataract, such as p.R12C, p.R21W/L, p.R49C, p.R54C, p.G98R, and p.R116C/H [6,25,26,27,28,29,30]. The associated cataract phenotypes in most patients correspond to nuclear or zonular types, and are commonly associated with microcornea, microphthalmia, and/or coloboma of the iris [6,26,27,30]. The recessive form of hereditary cataract is less common and has been identified in one Israeli family with a p.W9X nonsense mutation in the N-terminal domain of the CRYAA protein as well as in mice with the p.R54H mutation [31,32]. Khan et al. (2007) demonstrated that homozygous carriers of the p.R54C mutation have a congenital total cataract with microcornea, while heterozygous carriers have a bilateral isolated cataract [33]. The same mutation was found in another family, causing autosomal dominant congenital nuclear cataracts associated with microcornea [26]. Therefore, mutations in the *CRYAA* gene can lead to the development of congenital cataracts that differ in the nature of lens opacity and are often combined with microphthalmos and microcornea. α-crystallin also plays a crucial role in the embryonic development of the anterior segment of the eye in addition to its role in the lens. This was demonstrated in experiments with mice [34,35]. Based on this, it was suggested that, in addition to the lens, α-crystallin can also play an important role in the embryonic development of the anterior segment of the eye [27,30,36]. In the family we examined with the c.253C > T (p.L85F) mutation in the *CRYAA* gene, cataracts were classified as nuclear combined with microphthalmos and microcornea, consistent with previous reports. Other phenotypes associated with *CRYAA* variants can be more severe and are specifically associated with stop-loss variants that result in a C-terminal extension of the protein [37].

In one of the families examined, the mutation c.272_274delGAG (p.G91del) was identified in the *CRYBA1* gene. This deletion has been previously reported in several Chinese [2,21,38], European [20,39], and Iranian [40] families with congenital cataracts. The p.G91del mutation was found to be associated with autosomal dominant inheritance in all cases, but the phenotypic forms of the cataracts varied. While nuclear cataracts were observed in most cases, some patients had lamellar [20] or powdery [38] cataracts. In a large Chinese family, four patients with the p.G91del mutation had nuclear cataracts associated with esotropia and nystagmus, but the authors concluded that these symptoms were unlikely to be linked to the mutation [2]. The authors of the same study reported that the p.G91del mutation led to reduced expression of the CRYBA1 protein, which was observed in both capsule samples from patients and cell cultures. Other studies also investigated the functional role of this mutation. Structural studies revealed a decrease in the solubility of the mutant protein [20], and it was found that deletion of the G91 amino acid residue, which is located in the edge chain in β-sheets, can destabilize the protein [41]. Li et al. (2019) [2] confirmed that the deletion of this amino acid residue in the CRYBA1 protein can decrease protein stability, leading to its misfolding and denaturation in the lens structure. Thus, although the p.G91del mutation does not cause a frameshift or amino acid substitution, it has a clear pathological effect on protein function, leading to cataract development. In the family we examined, patients with the p.G91del mutation had zonular cataracts, according to their medical records.

The *GJA8* and *GJA3* genes are responsible for encoding connexin 50 and connexin 46 proteins, respectively. These proteins form connexons, which as previously mentioned are intercellular gap junctions. The rapid movement of potassium ions and other small molecules between cells is a crucial process for the normal functioning of the lens. This is because the lens lacks blood and lymphatic vessels as well as nerve fibers, and its nutrition is dependent on diffusion or active transport of nutrients, oxygen, and other substances through the capsule from the intraocular fluid. Mutations in connexins can disrupt the formation of gap junctions and channels, leading to the development of congenital cataracts [7].

Connexin 50 (Cx50) is an integral protein consisting of 432 amino acids; it plays a critical role in the formation of gap junctions, which facilitate the rapid movement of potassium ions and other small molecules between cells in the lens [42]. This is essential for the normal functioning of the lens, which lacks blood and lymphatic vessels as well as nerve fibers, and obtains nutrients and oxygen through diffusion or active transport via the intraocular fluid. More than 60 pathogenic mutations have been identified in the *GJA8* gene worldwide, making it a key player in the development of congenital cataracts. In our patient sample, the *GJA8* gene contained the highest number of pathogenic variants. Two unrelated families with autosomal dominant cataracts were found to carry the previously described missense mutation c.68G > C (p.R23T), while two other families had previously undescribed variants: c.133_142del deletion (p.W45Sfs*72) and the missense variant c.179G > A (p.G60D). In one patient with autosomal recessive cataracts, two compound heterozygous variants were identified—c.143A > G (p.E48G) and c.741T > G (p.I24M).

The p.R23T (rs80358203) mutation in the *GJA8* gene was previously identified in an Iranian family with autosomal dominant congenital progressive nuclear cataracts [11]. This mutation’s pathogenic effect on the function of connexin 50 was later confirmed. It alters the amino acid at position 23, changing the positively charged arginine to threonine, an uncharged polar amino acid, leading to disruption of the intracellular transport of the protein and its localization in the cytoplasm instead of the cell membrane. This leads to a loss of gap junction conductivity and the development of cataracts. The p.R23T mutation also inhibits co-expressed wild-type Cx50 and has been shown to have an inhibitory effect on Cx50 function [43]. Studies have shown that R23 is involved in the interhelix H-bond with Y151 in the side chain (TM3), and this bond is destroyed upon mutation of p.R23T, potentially destabilizing interhelix interaction but not inducing intramolecular steric collision [44]. Furthermore, this mutation leads to acidification of the surface electrostatic potential of the Cx50 molecule. Thus, the pathogenic role of the c.68G > C (p.R23T) mutation is well established and leads to the loss of Cx50 function and the development of cataracts due to the loss of Cx50-mediated intercellular communication.

The novel ten-nucleotide deletion c.133-142del (p.W45SfsX72) in the *GJA8* gene, identified in our study, results in a frameshift and the formation of a premature stop codon. Tryptophan-45, located in the border region between the first transmembrane domain (M1) and the first extracellular loop (E1) of connexin 50, is highly conserved among different animal species. Previously, the missense mutation c.134G > C (p.W45S) was found in the 45th amino acid position of Cx50 in patients with autosomal dominant congenital cataracts. This mutation was identified in an Indian family, and all affected members of the family exhibited jellyfish-like cataracts that were associated with microcornea [45]. In addition, another missense mutation in the adjacent 44th codon of the cDNA gene (p.V44E) was identified by other authors [46] that also leads to cataracts associated with microcornea. It has been established that both of these mutations cause Cx50 proteins to be correctly localized on cell membranes but unable to form functional gap junction channels, resulting in a complete loss of conductivity. In addition, these mutations have an inhibitory effect on the wild-type allele of the gene [44]. The patient with this mutation who we examined presented a congenital anterior polar cataract, but did not exhibit microcornea.

We have identified a previously unreported missense variant, c.179G > A (p.G60D), that affects the evolutionarily conserved glycine at position 60 in the extracellular loop E1 of the Cx50 protein [47]. The E1 loop is essential for the docking of connexons and for creating the electrical tension required to close gap junction pores. Mutant proteins can disrupt the normal interaction between connexons, leading to reduced intercellular channel conductivity. Previous studies have demonstrated the effect of changes in charge in the E1 loop on gap junction closure [48]. Our in silico analysis indicates that the p.G60D substitution is functionally significant and has a damaging effect on the protein’s structure and function. The substitution of glycine, an uncharged amino acid, with aspartic acid, a monoaminodicarboxylic acid that carries a negative charge in solution, could have an impact on the processes described above. We believe that the c.179G > A nucleotide substitution and the corresponding p.G60D amino acid substitution are likely pathogenic mutations that can lead to the development of congenital cataract. It is noteworthy that in the patient we examined with this mutation, zonular cataracts were combined with microphthalmos and microcornea.

The newly identified missense variant c.143A > G (p.E48G) is located in the E1 domain of the Cx50 protein and was found in a patient with autosomal recessive congenital cataracts. Notably, a mutation at the same 48th codon was previously reported—p.E48K, where negatively charged glutamic acid is replaced with positively charged lysine. This mutation was identified in patients with autosomal dominant cataracts in a Pakistani family, and the cataracts were described as zonular nuclear [49]. Studies [50] have demonstrated that p.E48K disrupts gap junction conductance and inhibits the conductance of the gap junction channel of co-expressed Cx50 wild type, but not the conductance of the wild-type hemichannel. It was also shown in [44] that p.E48K leads to the loss of the H-bond with R76 in the second transmembrane domain (TM2) and significantly alters the protein’s surface electrostatic potential, without causing any steric collisions. The authors suggested that this mutation might destabilize the Cx50 monomer by disrupting its folding (α-helix conformation), ultimately affecting oligomerization and intracellular protein transfer, leading to the loss of gap junction activity. In contrast, the p.E48G variant we identified replaces the negatively charged glutamic acid with glycine, a polar uncharged amino acid. This change in the surface electrostatic potential of the protein could also have pathological effects. Predictive in silico programs support the pathogenic role of the nucleotide variant c.143A > G(p.E48G). However, since this variant was found in a compound heterozygous state in a cataract patient and was also present in the patient’s healthy mother, it does not seem to have a dominant-negative effect.

The compound heterozygous state in the *GJA8* gene that included the previously described missense variant c.741T > G (p.I247M) was found in a patient. This variant was first identified in a Russian family with autosomal dominant cataracts [51] and was later found in a family from Germany in a patient with congenital diffuse cataracts, as well as in her healthy mother [52]. Despite analyzing a number of other candidate genes, no other genetic change responsible for cataracts in the patient was identified [52]. The nucleotide variant c.741T > G was not found in a control population sample of 179 people. However, a number of cellular and electrophysiological studies showed no pronounced effect of the p.I247M amino acid substitution on the formation of gap junctions and their electrical conductivity. The in silico predictive programs for this nucleotide substitution were contradictory. Thus, the role of the p.I247M missense variant in the GJA8 gene still remains unclear. Although there are three known unrelated families in which congenital bilateral cataract is associated with the heterozygous carriage of p.I247M, the variant is also found in healthy individuals, albeit with a low frequency, and there is evidence that indicates its neutral nature. The cataracts in all examined patients who carry p.I247M had similar phenotypes and were described as pulverulent, diffuse, or zonular. Therefore, the nucleotide substitution c.741T > G (p.I247M) can still be classified as a variant with an uncertain value, but it may be a pathogenic mutation with incomplete penetrance, depending on some additional genetic or epigenetic factors. It is important to note that codon 247 is part of the last intracellular domain of the protein and very few of the identified cataract-associated connexin mutations lie at the COOH terminus. Additionally, the amino acids isoleucine and methionine belong to the group of neutral amino acids with a non-polar side chain. The removal of the COOH terminus of Cx50 causes only modest effects on voltage-dependent blocking of the gap junction channel [53,54]. In summary, the available data suggest that the pathogenicity of the p.I247M missense variant in the *GJA8* gene is still unclear. Although it has been found in families with congenital bilateral cataracts [49,50], it is also present in healthy individuals. Additional research is necessary to determine its role in the development of cataracts and whether it is a pathogenic mutation with incomplete penetrance.

Connexin 46 (Cx46) and Cx50 are transmembrane proteins that consist of four transmembrane domains, two extracellular loops, one intracellular loop, and cytoplasmic NH2- and COOH-terminal domains [55,56]. Autosomal dominant cataracts are caused by more than 40 different missense mutations in the *GJA3* gene [https://cat-map.wustl.edu/home/cat-map-variant-file/ (accessed on 20 February 2022)]. These mutations are predominantly located in the NH2-terminal domain of the protein, transmembrane domains M1 and M2, and extracellular domains E1 and E2. However, mutations in the cytoplasmic loop CL have been identified in isolated cases, and no mutations have been described in transmembrane domains M3 and M4. In our study, we identified a previously undescribed deletion, c.del1126_1139 (p.D376Qfs*69), in the *GJA3* gene, which leads to a frameshift, a change in the splicing site, and an elongation of the COOH-terminal domain by 26 amino acids. This deletion was detected in three patients from the same family with autosomal dominant cataracts. The C-terminal domain of connexin 46 is subject to post-translational modifications, such as phosphorylation, which play a crucial role in the regulation of assembly and modulation of the physiological properties of channels [57,58]. Mackay et al. (1999) [59] previously described a single nucleotide insertion (1137insC (fs380)) that leads to an elongation of the COOH terminus of the Cx46 molecule by 31 amino acids and disrupts the formation of intercellular gap junctions in a Chinese family with autosomal dominant congenital zonular powdery cataracts. Later it was found that this mutation leads to disruption of the formation of intercellular gap junctions [60]. Another mutation, insertion c.1361_1362insC (p.A397Gfs71), leading to an elongation of the COOH terminus of connexin 46, was described in patients from China with congenital cataracts [61]. Based on these findings, we hypothesize that the c.del1126_1139 (p.D376Qfs*69) deletion identified in our study may also negatively impact the assembly of connexons, leading to disrupted formation of intercellular ion channels and ultimately a disruption in the interaction of lens fibers.

Studies have demonstrated that mutations in the *GJA3* gene primarily lead to congenital cataracts, with varying phenotypes including nuclear and zonular powdered cataracts [62,63,64,65,66]. Similarly, the cataracts observed in our patients with the p.D376Qfs*69 mutation were congenital and categorized as zonular cataracts, which aligns with the findings of the previously mentioned authors.

Our findings provide valuable insights into the spectrum of mutations in the studied genes and their associated phenotypes in hereditary cataracts. This knowledge enhances our understanding of the genogeography of the disease, which is critical for developing optimal DNA diagnostic and genetic counseling strategies in the specific region under investigation. To gain a deeper understanding of the pathogenesis of cataracts resulting from mutations in these genes, further functional investigations are needed to examine the impact of both known and novel nucleotide variants. Additionally, it is crucial to identify the genetic and epigenetic factors that influence the penetrance of pathogenic mutations and the corresponding clinical phenotypes of cataracts.

## Figures and Tables

**Table 1:** Evaluation of the clinical significance of the identified single nucleotide substitutions in the *CRYAA*, *CRYGD*, *CRYBA1*, *GJA8*, and *GJA3* genes based on the in silico analysis results and the frequency of the minor allele.

Gene	Nucleotide Substitution (Amino Acid Substitution)	SIFT	Poly-Phen2	LRT_Score	Mutation Taster	Mutation Assessor	FATHMM	PROVEAN	MetaLR	M-CAP	CADD	MAF «1000 Genomes» and ExAc	Clinical Relevance
*CRYAA*	c.253C > T (p.L85F)	0.002 (D)	0.999 (D)	0.0 (D)	1 (D)	0.894 (M)	0.937 (D)	0.650 (D)	0.961 (D)	0.940 (D)	27.5 (M)	-	likely pathogenic (PM1, PM2, PP1, PP3)
	c.291C > G (p.H97Q)	0.121 (T)	0.458 (P)	0.0 (D)	0.997 (D)	0.263 (L)	0.920 (D)	0.733 (D)	0.814 (D)	0.749 (D)	20.2 (M)	0.00000832 (ExAc)	likely pathogenic (PM1, PM2, PP2,PP3
*CRYGD*	c.130A > G (p.M44V) rs61731517	0.37 (T)	0.063 (B)	0.0 (D)	0.995 (D)	0.025 (N)	0.707 (T)	0.391 (N)	0.097 (T) 0.364	-	9.771 (M)	0.006 (1000 G-all) 0.008 (ExAc-all)	likely benign (BS2, BP4, PP4)
	c.376G > A (p.V126M) rs150318966	0.03 (D)	0.999 (D)	0.0 (D)	1.0 (D)	0.983 (H)	0.860 (D)	0.580 (D)	0.830 (D)	0.125 (D)	32.0 (M)	0.006 (1000 G-all) 0.002 (ExAc-all)	likely benign (BS2, PP4)
*CRYBA1*	c.272_274delGAG (p.G91del)	-	-	-	-	-	-	-	-	-	-	-	pathogenic (PS3, PM2, PM4, PP1)
*GJA8*	c.133_142del (p.W45Sfs*72)	-	-	-	-	-	-	-	-	-	-	-	pathogenic (PVS1, PM1, PM2)
	c.68G > C (p.R23T)	0.002 (D)	1.0 (D)	0.0 (D)	1.0 (D)	0.745 (M)	0.992 (D)	0.686 (D)	0.977 (D)	0.900 (D)	25.4 (M)	-	pathogenic (PM1, PP1, PP3, PS3)
	c.179G > A (p.G60D)	0.0 (D)	1.0 (D)	0.0 (D)	1.0 (D)	0.944 (M)	0.998 (D)	0.935 (D)	0.995 (D)	0.934 (D)	27.8 (M)	-	likely pathogenic (PM1, PM2, PP3)
	c.143A > G (p.E48G)	0.0 (D)	1.0 (D)	0.0 (D)	1.0 (D)	0.941 (H)	0.996 (D)	0.935 (D)	0.997 (D)	0.993 (D)	26.8 (M)	--	likely pathogenic (PM1, PM2, PM5, PP3)
	c.741T > G (p.I247M) rs80358202	0.154 (T)	0.468 (P)	0.0 (D)	0.00 (A)	0.170 (N)	0.976 (D)	0.084 (N)	0.851 (D)	-	0.023 (M)	0.0008 (1000 G-all) 0.003 (ExAc-all)	uncertain significance (PS4, PP4, BS3)
*GJA3*	c.1126_1139del (p.D376Qfs*69)	-	-	-	-	-	-	-	-	-	-	-	pathogenic (PM4, PM2, PP1, PP4)
	c.398G > A (p.R133Q) rs149933083	0.02 (D)	0.708 (P)	0.01 (U)	1.0 (D)	0.801 (M)	0.978 (D)	(0.672) (D)	0.884 (D)	0.302 (D)	22.4 (M)	0.001 (1000 G-all) 0.004 (ExAc-all)	likely benign (BS2, PP4)
	c.231C > T (p.Phe77=) rs143508620	-	-	-	-	-	-	-	-	-	9.085 (Low)	0.002 (1000 G-all) 0.003 (ExAc-all)	likely benign (BS2, PP4)

**Table 2:** Mutations in the *CRYAA*, *CRYBA1*, *GJA8* and *GJA3* genes and corresponding clinical variants in patients with hereditary cataracts.

Gene	Nucleotide Substitution (Amino Acid Substitution)	Number of Unrelated Families, Ethnic Group	Inheritance Type	Cataract Phenotype
*CRYAA*	c.253C > T (p.L85F)	1, Tatar	AD	patients 1, 2—nuclear cataract, combined with microphthalmos and microcornea, later—nystagmus and convergent strabismus
	c.291C > G (p.H97Q)	2, Bashkir	family 1—? family 2—AД	patient 1—posterior polar cataract patient 2—cataract type—? + lens subluxation
*CRYBA1*	c.272_274delGAG (p.G91del)	1, Russian	AD	patients 1, 2, 3—zonular cataract
*GJA8*	c.68G > C (p.R23T)	2, Russian	AD	patient 1—punctate cataract patient 2—nuclear cataract
	c.133_142del (p.W45Sfs*72)	1, Bashkir	AD	anterior polar cataract
	c.179G > A (p.G60D)	1, Russian	AD	zonular cataract associated with microphthalmos and microcornea
	c.143A > G (p.E48G) +c.741T > G (p.I247M)	1, Russian	AR	zonular cataract
*GJA3*	c.1126_1139del (p.D376Qfs*69)	1, Tatar	AD	zonular cataract

Note: the first identified variants of the studied genes and corresponding proteins are highlighted in gray.

## Data Availability

The data presented in this study are available on request from the corresponding author. The data are not publicly available due to privacy and ethical restrictions.
